# Recombinant FOXN1 fusion protein increases T cell generation in aged mice

**DOI:** 10.21203/rs.3.rs-2557067/v1

**Published:** 2023-02-08

**Authors:** Jin Zhao, Zhenzhen Zhang, Kuan Chen Lai, Laijun Lai

**Affiliations:** University of Connecticut; University of Connecticut; University of Connecticut; University of Connecticut

**Keywords:** FOXN1, Thymus, thymic epithelial cells, T cell generation, aged mice

## Abstract

**Background:**

Although the thymus continues to export T cells throughout life, it undergoes a profound involution/atrophy with age, resulting in decreased numbers of T cells in the older adult, which has direct etiological linkages with many diseases. T cell development in the thymus is dependent on the thymic microenvironment, in which thymic epithelial cells (TECs) are the major component. However, TECs undergo both a qualitative and quantitative loss during aging, which is believed to be the major factor responsible for age-dependent thymic atrophy. FOXN1 plays a critical role in TEC development and adult TECs maintenance. We have previously reported that intrathymic injection of a recombinant (r) protein containing FOXN1 and a protein transduction domain increases the number of TECs in mice, leading to enhanced thymopoiesis. However, intrathymic injection may not be an ideal choice for clinical applications. In this study, we produce a rFOXN1 fusion protein containing the N-terminal of CCR9, FOXN1 and a protein transduction domain.

**Results:**

We show here that, when injected intravenously into aged mice, the rFOXN1 fusion protein migrates into the thymus and enhances thymopoiesis, resulting in increased T cell generation in the thymus and increased number of T cells in peripheral lymphoid organ.

**Conclusions:**

Our results suggest that the rFOXN1 fusion protein has the potential to be used in preventing and treating T cell immunodeficiency in the older adult.

## Background

With modern advances in medicine, we are living longer than ever before. Although the news that our average lifespan is increasing is good, this fact presents new challenges both to the individual and to society. Advanced age is often accompanied by chronic disease, which not only negatively affects an individual’s quality of life, but also has a significant impact on our society since meeting the medical needs of the growing population of the older adult is a huge challenge (1, 2).

Aging affects several organ systems, and the immune system is one of the systems most significantly affected (1–5). The deleterious effects of aging on the immune system are well recognized as they lead to increased susceptibility to infection and cancer. In addition, the efficiency of vaccination is significantly reduced, limiting preventative prophylaxis.

The thymus is a specified immune organ that provides an inductive environment for the generation of T cells that play a critical role in the adaptive immune system. Although the thymus continues to export T cells throughout life, it undergoes a profound atrophy with age, a process termed thymic involution, resulting in decreased numbers and functional capacity of T cells in the older adult, which has direct etiological linkages with many diseases (1–5). Furthermore, T cell immune deficiency in the older adult is exacerbated when the immune system is insulted by chemotherapy, radiotherapy, infections (e.g. HIV), and preparative regimens for foreign tissue or organ transplants. Therefore, restoring thymus function in the older adult has important implications (1–5).

T cell development in the thymus is dependent on the thymic microenvironment, in which thymic epithelial cells (TECs) are the major component (6–11). Despite the importance, TECs undergo both a qualitative and quantitative loss over time, which is believed to be the major factor responsible for age-dependent thymic atrophy/involution (1–5). It is generally acknowledged that FOXN1 is a pivotal regulator for TEC development (12–21). FOXN1 is expressed in fetal thymus and postnatal TECs and its expression is progressively downregulated with aging (16, 17, 21–24). FOXN1 is required not only for TEC development in fetal thymus, but also for maintenance of the postnatal thymus (16, 17, 19, 21–26).

We have previously reported that intrathymic injection (i.t.) of a recombinant (r) protein containing FOXN1 and a protein transduction domain embedded in the HIV transactivator of transcription (TAT) protein (amino acids 47–57) increases the number of TECs in mice that have undergone congenic or allogeneic hematopoietic stem cell transplantation (27). Consequently, these mice had enhanced thymopoiesis, an improved thymic output and an increased number of naïve T cells in the periphery (27). However, i.t. injection may not be an ideal choice for clinical applications.

It has been reported that chemokine CCL25 is highly expressed in thymic tissue, especially thymic stroma (28). CCR9 is the receptor for CCL25 (29). Unlike other CC chemokine receptors, CCR9 shows a strict specificity for its ligand CCL25 (28–30). It has been shown intramural injection of a fusion protein containing the N-terminal of CCR9 and IL-7 increased the content of IL-7 in the thymus as compared to injection of IL-7 alone (28).

In this study, we develop a rFOXN1 fusion protein that contains the N-terminal of CCR9, FOXN1 and TAT. We show here that, when injected intravenously (i.v.) into aged mice, the rFOXN1 fusion protein can migrate into the thymus and enhance T cell generation in the thymus, resulting in increased number of peripheral T cells. Our results suggest that the rFOXN1 fusion protein has the potential to be used in preventing and treating T cell immunodeficiency in the older adult.

## Results

### 1. Expression, purification, and characterization of rFOXN1 and control fusion proteins

**Figure 1:**
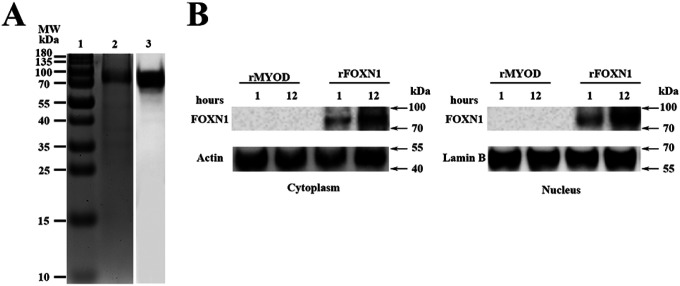
Characterization of the purified rFOXN1 fusion protein. (A) Gel and blot show purified rFOXN1 fusion protein; Lane 1: molecular weight markers; 2: Coomassie blue-stained SDS-PAGE; 3: Western blot with anti-FOXN1 antibody. (B) ESCs from NU/J nude mice were incubated with rFOXN1 or rMyoD fusion protein. Western blot analysis of FOXN1 protein from cytoplasmic and nuclear extractions. Actinand Lamin B were used as loading controls for cytoplasmic and nuclear proteins, respectively. (A, B) Data are representative of three independent experiments with similar results.

To produce rFOXN1 fusion protein, the extracellular domain of the murine *CCR9* and human *FOXN1* genes were connected by a flexible linker encoding (Gly4Ser)_2._ The *TAT* DNA was added to the C-terminal of the *FOXN1*. The entire DNA fragment of the *CCR9-FOXN1-TAT* was cloned into a prokaryotic expression vector pET-45b(+) that was then transformed into Rosetta 2 (DE3) bacteria. rFOXN1 fusion protein was purified from the bacteria. A relatively high purity of rFOXN1 fusion protein was obtained, as determined by Coomassie blue-stained SDS-PAGE (Figure 1A, lane 2). The identity of the protein was verified by Western blot using an anti-human FOXN1 antibody (Figure 1A, lane 3). For controls, a human MyoD fusion protein that contains murine CCR9 and human MyoD-TAT was similarly cloned, expressed and purified with the same system; the purified protein was verified by SDS-PAGE and Western blot using an anti-human MyoD antibody (data not shown).

We then determined whether the rFOXN1 fusion protein, when added into cultured cells, could translocate into the cytoplasm and nucleus of cultured cells. A mouse embryonic stem cells (ESCs) from NU/J nude mice that lack FOXN1 protein were cultured in the presence rFOXN1 or control rMyoD fusion protein. One or 12 hours later, nuclear and cytoplasmic protein extracts were prepared and subjected to Western blot analysis using the anti-FOXN1 antibody (27). The FOXN1 fusion protein was present in both the cytoplasm and nucleus after 1 and 12 hours, but more in nucleus after the longer incubation (Figure 1B). The data suggest that the rFOXN1 fusion protein can translocate from the cell surface into the cytoplasm and nucleus.

### 2. The rFOXN1 fusion protein can migrate into the thymus when injected peripherally

**Figure 2:**
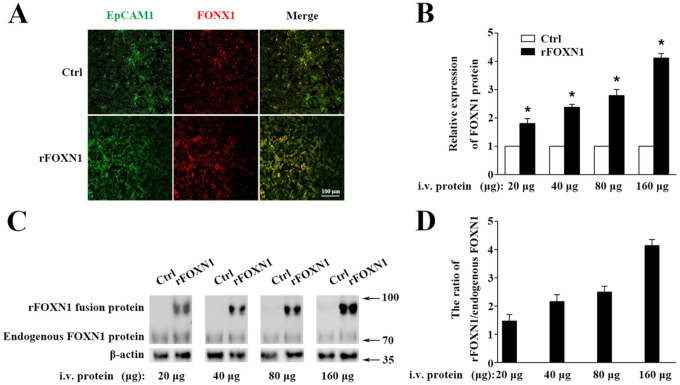
rFOXN1 fusion protein migrates into the thymus when injected peripherally. B6 mice were injected i.v. with graded doses of rFOXN1 fusion protein or rMyoD control protein (20, 40, 80, and 160 μg). One day later, the thymi were harvested and analyzed for FOXN1 expression levels by (A, B) immunofluorescence and (C, D) Western blot using anti-FOXN1 antibody. (A) Representative images of EpCAM1^+^ TECs (green color) and the expression of FOXN1 (red color). (B) Relative expression levels of FOXN1 in the thymus of rFOXN1 fusion protein- and control protein-treated mice. The expression levels of FOXN1 in the control protein-treated mice were defined as 1. (C) Representative images of the expression of endogenous mouse FOXN1 and human rFOXN1 fusion protein. (D) The ratio of human rFOXN1 fusion protein/endogenous mouse FOXN1 protein in rFOXN1-treated mice. (B and D) Data are shown as mean ± SD (n = 8 mice/group) and are representative of three independent experiments with similar results. Statistical significance determined by unpaired two-tailed Student’s t-test. * P<0.05 compared with control protein-treated mice.

To determine the ability of the rFOXN1 fusion proteinto travel to the thymus from the periphery, C57BL/6 (B6) mice were injected i.v. with graded doses of the rFOXN1 or control fusion protein(20, 40, 80, and 160 μg). One day later, the thymus was harvested. Frozen thymus sections were prepared and analyzed for the expression of FOXN1 by immunofluorescence. After staining with antibodies against FOXN1 and EpCAM1 (to identify TECs), the samples were observed under a confocal microscope. Since the anti-FOXN1 antibody reacts with both the endogenous mouse FOXN1 and the human rFOXN1 fusion protein, we detected the total expression levels of both FOXN1 proteins with immunofluorescence. As shown in Figure 2A and B, i.v. injection of the rFOXN1 fusion protein resulted in increased expression of FOXN1 in the TECs in a dose-dependent manner with more than 2-, 2.5- and 4-fold increase with 40, 80 and 160 μg rFOXN1 fusion protein, as compared with respective doses of control protein.

We then performed Western blot to confirm the expression levels of FOXN1. The lysis of the thymus tissues was analyzed by Western blot using the anti-FOXN1 antibody that also reacted with both mouse and human FOXN1. Since the molecular weight of the rFOXN1 fusion proteinis higher than that of endogenous mouse FOXN1, we could separate the expression levels of the two FOXN1 proteins with this method. As shown in Figure 2C and 2D, the expression levels of the endogenous mouse FOXN1 protein were not significantly changed after injection of graded doses of the FOXN1 or control fusion protein. In contrast, the expression levels of the rFOXN1 fusion proteinin the thymus were significantly increased in a dose-dependent manner after i.v. injection ofthe rFOXN1 fusion protein (Figure 2C and D). Similar trends were also observed whenCD45^−^EpCAM1^+^ TECs were purified from the thymus, and the cytoplasmic and nuclear fractions in the TECs were separated and analyzed for FOXN1 protein expression by Western blot (data not shown). Taken together, the results suggest that the rFOXN1 fusion protein can migrate into the thymus when injected peripherally.

### 3. The rFOXN1 fusion protein increases the number of total TECs and TEC subsets in aged mice

**Figure 3:**
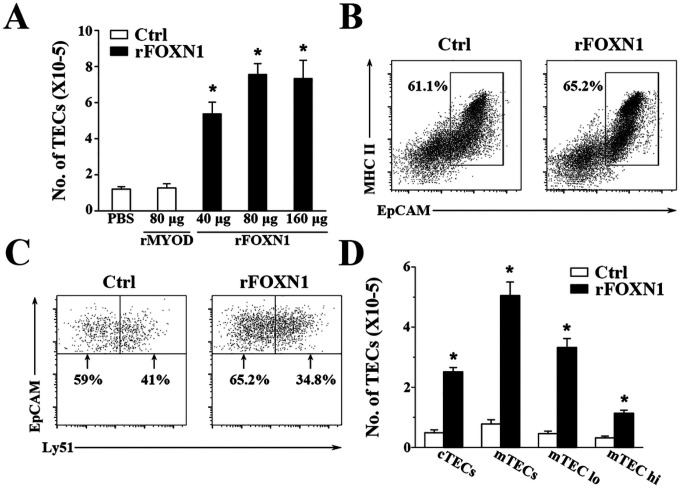
rFOXN1 fusion protein increases the number of total TECs and TEC subsets in aged mice. B6 mice (14-month-old) were injected i.v. with graded doses of rFOXN1 fusion protein (40, 80, and 160 μg), control rMyoD fusion protein (80 μg), or PBS at 6 day-intervals. Two months later, the thymi were harvested and analyzed by flow cytometry. (A) The number of total TECs (CD45^−^EpCAM^+^), and (B-D) the percentages and numbers of cTECs (CD45^−^EpCAM^+^MHC II^+^Ly51^+^), mTECs (CD45^−^EpCAM^+^MHC II^+^Ly51^−^), mTECs^lo^ and mTECs^hi^ TEC subsets from the 80 μg rFOXN1 and control fusion protein treated mice are shown. (B, C) Representative flow cytometric prfiles showing the percentage of (B) EpCAM^+^MHC II^+^ TECs in CD45^−^ thymic stromal cells and (C) Ly51^+^ cTEC and Ly51^−^ mTECs in CD45^−^EpCAM^+^MHC II^+^ TECs. (A and D) Data are shown as mean ± SD (n = 8 mice/group) and are representative of three independent experiments with similar results. Statistical significance was determined by (A) one-way ANOVA with Dunnett test and (D) unpaired two-tailed Student’s t-test. * P<0.05 compared with control rMyoD protein-treated mice.

We then determined the ability of the rFOXN1 fusion protein to increase the number of TECs in aged mice. Fourteen-month-old B6 mice were injected i.v. with graded doses of the rFOXN1 or control rMyoD fusion protein (40, 80, and 160 μg) at 6 day-intervals. PBS was also used as a negative control. Two months later, the thymi were harvested and the number of total TECs (CD45^−^EpCAM^+^) was analyzed by flow cytometry. rMyoD did not affect the number of total TECs at any dose, as compared to PBS treatment (Figure 3A and data not shown). In contrast, the numbers of total TECs in 40, 80, and 160 μg rFOXN1 fusion protein-treated mice were increased 3-, 4-, and 3.9-fold, respectively, above those in control rMyoD protein-treated mice (Figure 3A).

TECs can be broadly divided into cortical TECs (cTECs) and medullary TECs (mTECs) that are Ly51^+^UEA^−^ and Ly51^−^UEA^+^, respectively. The rFOXN1 fusion protein increased the numbers of both CD45^−^ EpCAM^+^MHC II^+^Ly51^+^ cTECs and CD45^−^EpCAM^+^MHC II^+^Ly51^−^ mTECs with a greater effect on mTECs, as compared with the rMyoD control protein (Figure 3B, C, D). Similar results were obtained when CD45^−^ EpCAM^+^MHC II^+^UEA^−^ cTECs and CD45^−^EpCAM^+^MHC II^+^UEA^+^ mTECs were analyzed (data not shown). mTECs can be further divided into mTECs^lo^ and mTECs^hi^ subsets based on the expression level of MHC II, and the former are considered as immature mTECs (31–33). As shown in Figure 3D, rFOXN1 fusion protein increased the number of both mTECs^lo^ and mTECs^hi^. Taken together, our results suggest that the rFOXN1 fusion protein treatment increases the number of total TECs and TEC subsets in aged mice.

### 4. The rFOXN1 fusion protein increases the number of total thymocytes and thymocyte subsets including early thymic progenitors (ETPs) in aged mice

**Figure 4:**
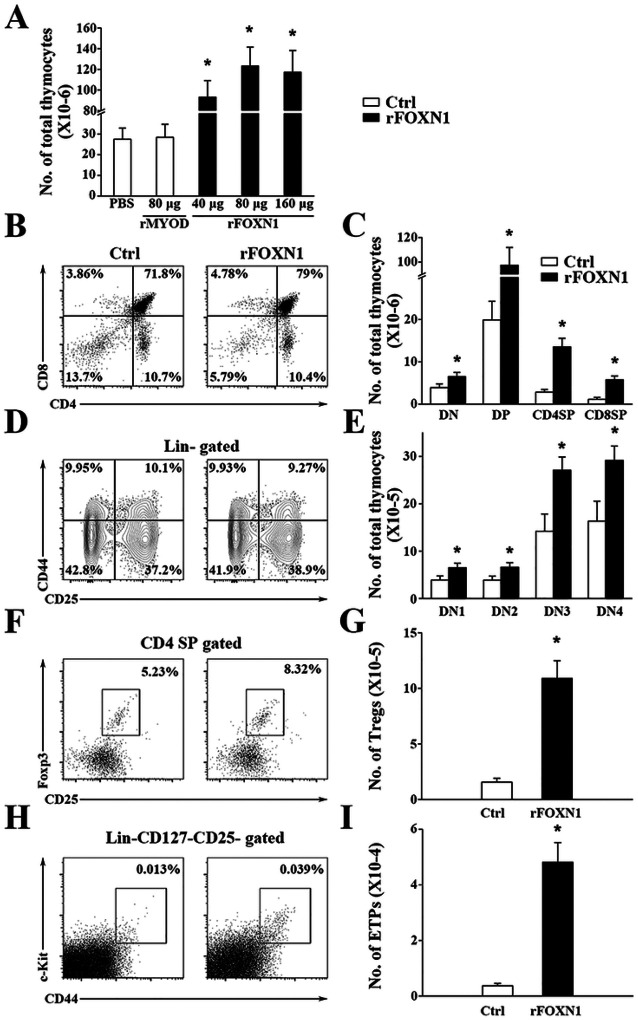
The rFOXN1 fusion protein increases the number of total thymocytes and thymocyte subsets in aged mice. B6 mice (14-month-old) were injected i.v. with graded doses of rFOXN1 or control rMyoD fusion protein at 6 day-intervals as in Figure 3. Two months later, the thymi were harvested and analyzed byow cytometry. (A) The number of CD45^+^ total thymocytes from all groups of mice. (B-I) The percentages and numbers of thymocyte subsets from the 80 μg rFOXN1 or MyoD fusion protein treated mice. (B) Representative flow cytometric prfiles showing the percentage of CD4 and CD8 DN, DP, and SP thymocytes (analyzing 10,000 cells per sample). (C) Number of CD4 and CD8 DP, CD4 SP and CD8 SP thymocytes. (D) Representative flow cytometric prfiles showing the percentage of DN1-DN4 thymocytes after gating on lin^−^ cells (one million cells per sample). (E) Number of DN1-DN4 thymocytes. (F) Representative flow cytometric prfiles showing the percentage of CD4^+^CD25^+^FoxP3^+^ Treg cells. (G) Number of the Treg cells. (H) Representative flow cytometric prfiles showing the percentage of lin^−^c-kit^+^IL-7Rα^−^CD44^+^CD25^−^ ETPs in total thymocytes (one million cells per sample). (I) Number of ETPs. (A, C, E, G, and I) Data are shown as mean ± SD (n = 8 mice/group) and are representative of three independent experiments with similar results. Statistical significance was determined by (A) one-way ANOVA with Dunnett test and (C, E, G, I) unpaired two-tailed Student’s t-test. * P<0.05 compared with control rMyoD protein-treated mice.

We have previously shown that the increased number of TECs in the murine rFOXN1 protein treated mice that have undergone hematopoietic stem cell transplantation subsequently increased the number of thymocytes (27). We therefore determined whether the human rFOXN1 fusion protein has similar effect on aged mice. Like the effect on TECs, a parallel dose-dependent increase in the number of CD45^+^ total thymocytes was observed; the numbers of total thymocytes in 40, 80, and 160 μg rFOXN1 fusion protein-treated mice were increased 3.3-, 4.3-, and 4.1-fold, respectively, above those in control rMyoD protein-treated mice (Figure 4A).

Thymocytes can be divided into four major subsets: CD4 and CD8 double negative (DN), double positive (DP), CD4 single positive (SP), and CD8 SP thymocytes. The rFOXN1 fusion protein decreased the percentages of DN thymocytes but increased the percentages of DP and CD8 SP thymocyte subsets (Figure 4B). Because the rFOXN1 fusion protein increased the number of total thymocytes, it increased the numbers of each thymocytes subsets including DN thymocytes (Figure 4C). DN thymocytes can be further divided into DN1 to DN4 subsets based on the expression of CD44 and CD25. rFOXN1 treatment did not significantly change the percentage of the subsets but increased the cell numbers of each subset (Figure 4D, E). Since natural regulatory T cells (Tregs) also develop in the thymus (34), we analyzed these cells. rFOXN1 treatment increased the percentage and number of CD4^+^CD25^+^FoxP3^+^ Tregs in the aged mice (Figure 4F, G).

The early thymic progenitor (ETPs) (lineage^−^ c-kit^+^ IL-7Rα^−^ CD44^+^CD25^−^) are considered to represent canonical intrathymic T-cell progenitors (35–37). rFOXN1 treatment increased both the percentage and number of ETPs (Figure 4H, I). Taken together, our data suggest that the rFOXN1 fusion protein treatment increases the number of all thymocyte subsets including ETPs.

### 5. The rFOXN1 fusion protein-treated aged mice have increased number of peripheral T cells

**Figure 5:**
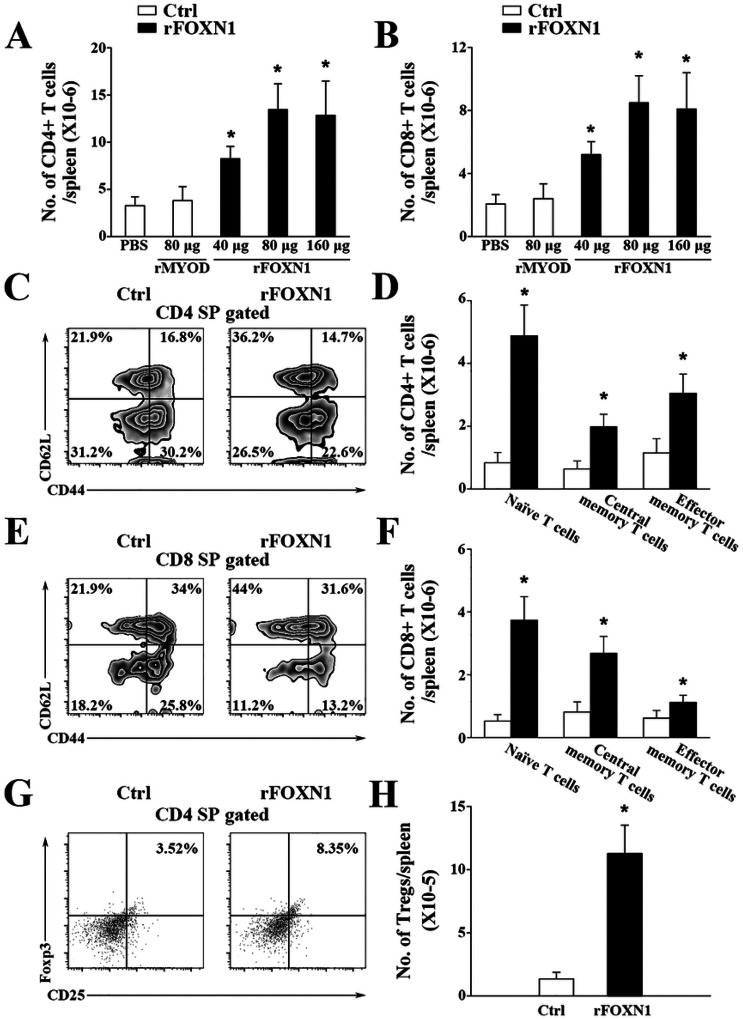
The rFOXN1 fusion protein-treated aged mice have increases number of peripheral T cells. B6 mice (14-month-old) were injected i.v. with graded doses of rFOXN1 or control rMyoD fusion protein at 6 day-intervals as in Figure 3. Two months later, the spleens were harvested and analyzed by flow cytometry. (A, B) The number of total (A) CD4^+^ and (B) CD8^+^ T cells from all groups of mice. (C-H) The percentages and numbers of CD4^+^ and CD8^+^ T cell subsets from the 80 μg rFOXN1 or MyoD fusion protein treated mice. (C, E) Representative flow cytometric prfiles showing the percentage of CD4 and CD8 CD44^lo^CD62L^hi^ naïve, CD44^hi^CD62L^lo^ effector memory, and CD44^hi^CD62L^hi^ central memory T cells. (D, F) The number of CD4 and CD8 naïve, effector memory, and central memory T cells. (G) Representative flow cytometric prfiles showing the percentage of CD4^+^CD25^+^FoxP3^+^ Tregs. (H) The number of the Tregs. (A, B, D, F, H) The data are shown as mean ± SD (n = 8 mice/group) and are representative of three independent experiments with similar results. Statistical significance was determined by (A, B) one-way ANOVA with Dunnett test and (D, F, H) unpaired two-tailed Student’s t-test. * P<0.05 compared with control rMyoD protein-treated mice.

We also determined whether the enhanced thymopoiesis in the rFOXN1 fusion protein-treated aged mice resulted in increased number of peripheral T cells. The spleens were harvested from the rFOXN1 or control fusion protein-treated aged mice 2 months later. rFOXN1 fusion protein treatment significantly increased the number of total CD4^+^ and CD8^+^ T cells; 40, 80, and 160 μg rFOXN1 fusion protein increased2.2-, 3.5-, and 3.4-fold CD4^+^ T cells and CD8^+^ T cells, respectively, above those in control rMyoD protein-treated mice (Figure 5A, B).

T cells can be divided into CD44^lo^CD62L^hi^ naïve, CD44^hi^CD62L^lo^ effector memory, and CD44^hi^CD62L^hi^ central memory T cells. rFOXN1 significantly increased the percentages and numbers of CD4^+^ and CD8^+^ naïve T cells (Figure 5C–F). Although rFOXN1 decreased the percentages of CD4^+^ and CD8^+^ effector memory and central memory T cells, because rFOXN1 fusion protein increased the number of total CD4^+^ and CD8^+^ T cells, it also increased the numbers of these T cells (Figure 5C–F).

We then analyzed CD4^+^CD25^+^FoxP3^+^ Tregs in the spleen. As shown in Figure 5G and 5H, rFOXN1 treatment increased the percentage and number of CD4^+^CD25^+^FoxP3^+^ Tregs in the aged mice. The increased numbers of CD4 and CD8 T cells, as well as Tregs were also similarly observed in lymph nodes and blood (data not shown). Collectively, our results suggested that the enhanced thymopoiesis in the rFOXN1 fusion protein-treated aged mice resulted in increased number of peripheral T cells.

## Discussion

Because CCR9 has a strict specificity for its ligand CCL25 and because CCL25 is highly expressed in thymic tissue, especially thymic stroma (28–30), we fused the N-terminal of murine CCR9 to human FOXN1. We have shown here that the rFOXN1 fusion protein can migrate into the thymus, especially TECs, after injected peripherally. The results suggest that CCR9 can direct FOXN1 protein to move to the thymus from the blood. Our data are consistent with the report that a fusion protein containing the N-terminal of CCR9 and IL-7, when injected peripherally, increased the content of IL-7 in the thymus, resulting in enhanced thymopoiesis (28).

We have also shown that i.v. injection of the rFOXN1 fusion protein increases the number of TECs in aged mice. Consequently, the aged mice have enhanced T cell generation in the thymus, leading to increased numbers of T cells in the peripheral lymphoid organs. Although the rFOXN1 fusion protein increases the number of TECs and thymocytes in a dose-dependent manner, administration of the protein at a 160 μg does not have higher effect than the 80 μg does. The data are consistent with our and other reports that the FOXN1 dosage needs to be tightly controlled (15–17, 23, 24, 27).

The human FOXN1 fusion protein can affect mouse TECs, likely because mouse and human FOXN1 proteins have a high homology. It has been reported that 85% of amino acids in human FOXN1 protein are identical to the murine form and both proteins are also identical in length (648 aa) (38).

Although age-dependent thymic involution/atrophy can be caused by both ETP and TEC degeneration, recent data have shown that a reduced expression or the deletion of a single gene, *FOXN1*, results in phenotypes similar to age-dependent thymic involution. Furthermore, overexpression of FOXN1 attenuates age-associated thymic involution (16, 23, 24). Our results that administration of the rFOXN1 fusion protein increases the number of TECs and thymopoiesis are consistent with these reports, suggesting that FOXN1 plays a critical role in maintaining adult TECs.

## Conclusions

We have shown that administration of the rFOXN1 fusion can increase the number of TECs, resulting in enhanced T cell generation in the thymus, leading to increased number of peripheral T cells. Our results suggest that the rFOXN1 fusion protein has the potential to be used in preventing and treating T cell immunodeficiency in the older adult.

## Methods

### Mice

C57BL/6 mice were obtained or purchased from NIA and Jackson Laboratory. The mice were used in accordance with protocols approved by the Institutional Animal Care and Use Committee of the University of Connecticut.

### Plasmid construction

The extracellular domain of the murine *CCR9* cDNA was amplified using the sense primer (primer 1) containing sequences homologous to the pET45b(+) vector, and antisense primer (primer 2) containing a linker encoding (Gly4Ser)2. The human *FOXN1-TAT* gene was amplified using the sense primer (primer 3) containing the linker and antisense primer (primer 4) containing TAT and 19 bases that are homologous to the pET45b(+) vector. The polymerase chain reaction (PCR) products of *CCR9* and *FOXN1-TAT* were combined and subjected to an overlap extension PCR with primers 1 and 4. The entire DNA fragment of the *CCR9-FOXN1-TAT* was cloned into the expression vector pET-45b(+) (Novagen, Gibbstown, NJ) using the In-Fusion Snap Assembly Kit (Clontech, Mountain View, CA) according to the manufacturer’s instructions. *CCR9-MyoD-TAT* gene was similarly cloned into the pET45b(+) vector. The plasmid constructs were verified by DNA sequencing.

Primers for *CCR9-FOXN1-TAT* pET45b(+) construction

**Table:** 

	Sequence 5′−3′
Primer 1	**CCATCACGTGGGTACCGGT**cccacagaactcacaagcct
Primer 2	CGACCCACCACCGCCCGAGCCACCGCCTCCGCTTGCAAACTGCCTGACATT
Primer 3	GGAGGCGGTGGCTCGGGCGGTGGTGGGTCGGTGTCGCTACCCCCGCCGCAGTCT
Primer 4	**tttctttaccagactcgag**CTATCAACGTCTACGTTGCCTTCG

Bold letters indicate the homologous to the pET45b(+) vector

### Recombinant protein purification and verification

The expression vector containing the *CCR9-FOXN1-TAT* or *CCR9-MyoD-TAT* gene was transformed into BL21(DE3) pLysS bacteria (Novagen). The bacteria were expanded in LB medium with ampicillin until OD600 reached 1.0–1.2. Protein expression was induced with 1mM isopropyl-β-D-1-thiogalactopyranoside (IPTG, Novagen) at 37 °C for 4 h. The rFOXN1 and rMyoD fusion proteins were purified and refolded using a Protein Refolding Kit (Thermo Scientific). Purified rFOXN1 and rMyoD fusion proteins were verified by SDS-PAGE, Coomassie Staining and Western blot. Protein was quantified using the Pierce^™^ BCA Protein Assay Kit (Pierce, Rockford, IL) according to the manufacturer’s instructions.

### SDS-PAGE and Western blot

Cytoplasmic and nuclear proteins were extracted from cells using the NE-PER^®^ Nuclear and Cytoplasmic Extraction Reagents (Thermo Scientific, Rockford, IL). Purified rFOXN1, rMyoD, cytoplasmic or nuclear proteins were loaded on a 10% SDS-PAGE, transferred to a polyvinylidene fluoride membrane, and then incubated with antibodies. The following antibodies were used: rabbit anti-FOXN1 (Thermo Scientific, or Fitzgerald, Acton, MA), rabbit anti-MyoD (Santa Cruz Biotechnology, Inc., Dallas, TX), and HRP conjugated secondary antibody. The membrane with proteins and antibodies was developed with Super Signal^®^ West Pico chemiluminescent Substrate (Thermo Scientific).

### Immunofluorescence

Immunofluorescence analysis of thymus tissues were performed as described (39). Briefly, tissues were incubated in 4% paraformaldeyde for 4 hours followed by incubation in 30% sucrose solution overnight. The tissues were embedded in OCT medium, snap frozen, and subsequently cut into 5 micrometer sections. The sections were stained with rabbit anti-FOXN1 (Thermo Scientific) and rat anti-mouse EpCAM1 antibody (Biolegend), followed by AlexaFluor-488-, and 546-conjugated goat anti-rabbit IgG, or goat anti-rat IgG (Invitrogen). The cells were observed under a Nikon A1R Spectral Confocal microscope (Nikon, Kanagawa, Japan).

### TEC isolation

The thymi were incubated at 37 °C in 0.01 (w/v) liberase (Roche, Nutley NJ) and 0.02% (w/v) DNAse I (Roche) with regular and gentle agitation as described (40). CD45^−^ cells were negatively selected by CD45 microbeads, and EpCAM^+^ cells were then positively selected by EpCAMmicrobeads (Miltenyi Biotec, San Diego).

### Flow cytometry

Single-cell suspensions of thymocytes and spleen cells were stained with one or more of the following fluorochrome-conjugated antibodies: CD4, CD8, CD3, CD25, CD44, CD62L, CD117, CD127, EpCAM1, Ly51, CD45, FoxP3, and I-A^b^ (BioLegend, BD Biosciences, San Jose, CA, or eBioscience, San Diego, CA). Fluorochrome-conjugated CD1 tetramers loaded with PBS57 were from National Institute of Health Tetramer Facility. ETPs were identified as lin IL-7Rα (CD127) c-kit (CD117) CD44 CD25 thymus cells. A cocktail of antibodies against TER-119, B220, CD19, IgM, Gr-1, CD11b, CD11c, NK1.1, TCRβ, CD3e, and CD8α was used to distinguish the lin^−^ cells. The samples were analyzed on a LSR II flow cytometer (BD Biosciences). Data analysis was done using FlowJo software (Ashland, OR).

### Statistical analysis

P-values were based on the two-sided Student’s t test. For comparing means of multiple groups, significance was determined using one-way ANOVA with Dunnett test. A confidence level above 95% (p<0.05) was determined to be significant.

## Data Availability

The datasets used and/or analyzed during the current study are available from the corresponding author on reasonable request.

## Abbreviations

TECs: thymic epithelial cells
rFOXN1: recombinant FOXN1 protein
TAT: transactivator of transcription
IPTG: isopropyl-β-D-1-thiogalactopyranoside
i.v.: intravenously
i.t.: intrathymic
ESC: embryonic stem cell
cTECs: cortical TECs
mTECs: medullary TECs
ETPs: early thymic progenitor
DN: double negative
DP: double positive
SP: CD4 single positive
Tregs: regulatory T cells
